# Model of fluid and solute shifts during hemodialysis with active transport of sodium and potassium

**DOI:** 10.1371/journal.pone.0209553

**Published:** 2018-12-28

**Authors:** Mauro Pietribiasi, Jacek Waniewski, Alicja Wójcik-Załuska, Wojciech Załuska, Bengt Lindholm

**Affiliations:** 1 Nalecz Institute of Biocybernetics and Biomedical Engineering Polish Academy of Sciences, Warsaw, Poland; 2 Department of Rehabilitation and Physiotherapy, Medical University of Lublin, Lublin, Poland; 3 Department of Nephrology, Medical University of Lublin, Lublin, Poland; 4 Renal Medicine and Baxter Novum, Karolinska Institutet, Stockholm, Sweden; Delft University of Technology, NETHERLANDS

## Abstract

**Background:**

Mathematical models are useful tools to predict fluid shifts between body compartments in patients undergoing hemodialysis (HD). The ability of a model to accurately describe the transport of water between cells and interstitium (*J*_*v*,*ISIC*_), and the consequent changes in intracellular volume (ICV), is important for a complete assessment of fluid distribution and plasma refilling. In this study, we propose a model describing transport of fluid in the three main body compartments (intracellular, interstitial and vascular), complemented by transport mechanisms for proteins and small solutes.

**Methods:**

The model was applied to data from 23 patients who underwent standard HD. The substances described in the baseline model were: water, proteins, Na, K, and urea. Small solutes were described with two-compartment kinetics between intracellular and extracellular compartments. Solute transport across the cell membrane took place via passive diffusion and, for Na and K, through the ATPase pump, characterized by the maximum transport rate, *Jp*_*MAX*_. From the data we estimated *Jp*_*MAX*_ and two other parameters linked to transcapillary transport of fluid and protein: the capillary filtration coefficient *Lp* and its large pores fraction *α*_*LP*_. In an Expanded model one more generic solute was included to evaluate the impact of the number of substances appearing in the equation describing *J*_*v*,*ISIC*_.

**Results:**

In the baseline model, median values (interquartile range) of estimated parameters were: *Lp*: 11.63 (7.9, 14.2) mL/min/mmHg, *α*_*LP*_: 0.056 (0.050, 0.058), and *Jp*_*MAX*_: 5.52 (3.75, 7.54) mmol/min. These values were significantly different from those obtained by the Expanded model: *Lp*: 8.14 (6.29, 10.01) mL/min/mmHg, *α*_*LP*_: 0.046 (0.038, 0.052), and *Jp*_*MAX*_: 16.7 (11.9, 25.2) mmol/min. The relative RMSE (root mean squared error)averaged between all simulated quantities compared to data was 3.9 (3.1, 5.6) %.

**Conclusions:**

The model was able to accurately reproduce most of the changes observed in HD by tuning only three parameters. While the drop in ICV was overestimated by the model, the difference between simulations and data was less than the measurement error. The biggest change in the estimated parameters in the Expanded model was a marked increase of *Jp*_*MAX*_ indicating that this parameter is highly sensitive to the number of species modeled, and that the value of *Jp*_*MAX*_ should be interpreted only in relation to this factor.

## Introduction

Fluid management is one of the principal objectives of the hemodialysis (HD) treatment. During the last decade there has been a shift among nephrologists, from removal of uremic toxins to control of fluid overload and preservation of optimal fluid distribution between different body compartments as the prime targets of HD, thus putting “volume first”[1, 2].

Mathematical models are useful tools to provide estimates of the possible results of an HD session for specific treatment settings—how much fluid will be removed and from where—allowing a personalized approach to HD therapy [3, 4]. Particularly interesting is the modelling of fluid shifts between interstitial and vascular spaces, and the transcapillary transport phenomena associated, which are at the basis of the so-called *plasma refilling*, one of the main mechanisms of fluid homeostasis preservation which are activated during water removal by HD [5–7], which has been modeled extensively [7–12].

However, the distribution of fluid between interstitial and intracellular spaces determines how much fluid is available in the interstitium to participate in a plasma refilling flow, and thus it should be taken into account in order to provide an accurate description of the refilling process. The balance of osmotically active small solutes between intra- and extracellular fluid, being the main driving force for the transport of water across the cellular membrane, must be properly described as well for a correct assessment of the latter. The problem of modelling electrolytes balance during HD has been long discussed with different scope and approaches, from lumped-parameters models with varying degrees of complexity [13–19] to finite elements models of the exchange within the dialyzer [20]. Some of the more recent models proposed the simultaneous description of multiple solutes [15, 18], albeit with a simplified view on transcapillary transport; moreover, very few examples were found of models assessing the contribution of the Na/K ATPase to the kinetics of total osmolarity during HD [14, 21, 22]. The action of these proteins, responsible for the active transport of some ionic species (in primis sodium and potassium) across the cell membrane, is extremely important to the preservation of the homeostasis in the organism, especially in conditions when the physiological equilibrium is perturbed, for instance during HD.

To assess the effect of such perturbations on the behavior of the active transport mechanism of sodium and potassium is difficult, especially in a clinical setting. Expressing the contribution of the Na/K pump with a simple parameter in a mathematical model might be a step in that direction. To this purpose, in this study we propose a model that describes the distribution of fluid in the three main body compartments (intracellular, interstitial and vascular) during HD, paired with the transport kinetics of both large and small solutes with an explicit description of the active transport for sodium and potassium. The mass exchange between plasma and interstitial fluid, including protein transport, plasma refilling and lymphatic absorption, that were detailed through an implementation of the 3-pore model of the capillary wall, were already described in a previous paper [12]. The action of the Na/K ATPase pump was summarized with a single parameter, the maximum rate of active transport on a whole-body level (*Jp*_*MAX*_).

The small solutes included in the model are sodium and potassium, chosen by virtue of their high concentration in extra- and intracellular fluids, respectively, and urea, because of its high concentration in HD patients. Another factor in choosing the chemical species to be modeled was the availability of clinical data to be used for the validation of the results. A major point of focus was to strike a good compromise between simplicity of implementation (and small number of parameters) and physiological accuracy. Three parameters of the model (including *Jp*_*MAX*_) were estimated by fitting the model to clinical data measured in standard HD sessions, with the purpose of demonstrating the model’s ability to simulate individual patient’s profiles and to obtain estimates on commonly unmeasurable quantities.

## Methods

### Clinical data

The data were collected in a previous study [23]. Twenty-three end-stage renal disease patients underwent thrice-weekly standard HD at the Lublin Medical University in Lublin, Poland. Pre-dialytic interval was 3 days for the first session, and 2 days for the others. Because of similar pre-dialytic status and modelling results in the last two sessions of the week, in this article only the first and last session will be described (HD1 and HD3).

The patients were 8 males and 15 females, with median age of 66 years, ranging from 38 to 84 years. The median preceding time on dialysis was 12 years, with range 1 to 32. Six patients had diabetes, but no discernible difference in the pre-dialysis fluid and solute status compared to the other patients. Two HD1 sessions and one HD3 session were excluded due to measurement artifacts in the data of relative blood volume. Written informed consent was obtained from each patient and the study was approved by the Ethical Committee of the Lublin Medical University.

The treatment settings are reported in Table 1. Ultrafiltration, blood and dialysis fluid flows were constant during each session. Dialysis fluid flow was the same in all sessions, 500 mL/min.

**Table 1:** Characteristics of the treatment, and of the patients (measured before each HD session) reported as median (quartiles). Dialysances were calculated from the concentrations at the inlet and outlet of the dialysate circuit, for each patients [24].

	HD1	HD3
Session length (min)	238 (234, 247)	240 (236, 253)
Blood flow (mL/min)	265 (230, 320)	280 (240, 320)
Ultrafiltration rate (mL/min)	11.5 (9.6, 13.6)*	9.2 (6.7, 10.0)
Body weight (kg)	67.3 (57.4, 80.2)*	69.7 (58.2, 78.8)
ECV (L)	16.3 (14.4, 19.3)*	15.5 (13.4, 17.5)
ICV (L)	14.5 (12.1, 17.0)	15.0 (11.8, 17.9)
Plasma volume (L)	3.2 (2.8, 3.4)*	3.0 (2.6, 3.3)
Fluid overload (L)	3.0 (2.0, 3.7)*	2.1 (1.1, 2.6)
MAP (mmHg)	93.0 (79.9, 110.5)	90.3 (80.7, 99.0)
Haematocrit (%)	31.5 (29.2, 32.8)	32.1 (29.8, 33.6)
Serum total protein (g/dL)	6.5 (6.2, 6.7)*	6.6 (6.4, 6.8)
Sodium (mmol/L)	140.0 (138.5, 142.5)	140.0 (137.0, 142.0)
Potassium (mmol/L)	5.6 (5.4, 6.1)	5.4 (5.1, 6.1)
Glucose (mmol/L)	4.7 (4.0, 5.9)	4.7 (4.0, 5.5)
Urea (mmol/L)	55.2 (44.9, 67.6)	46.2 (38.7, 52.1)
Urea clearance (mL/min)	220.9 (139.6, 232.4)	201.3 (195.5, 226.4)
Potassium dialysance (mL/min)	153.7 (129.5, 176.5)	146.9 (120.7, 169.2)
Dialysis fluid sodium (mmol/L)	142.0 (140.8, 144.0)	142.0 (141.0, 143.0)
Dialysis fluid potassium (mmol/L)	3.0 (2.1, 3.1)	2.9 (2.1, 3.1)

*ECV*–extracellular volume, *ICV*–intracellular volume, *MAP*–mean arterial pressure;

* p-value < 0.001 vs. HD3.

Body Composition Monitor (BCM, Fresenius, Bad Homburg, Germany) was used to assess the fluid volume of body compartments via bioimpedance spectroscopy. Plasma solutes concentrations (urea, glucose, sodium, potassium, total protein) were measured (Siemens Advia 1800) from blood samples collected before, after and at the beginning of every hour during HD (Table 1). Fresenius CritLine was used to estimate online blood haematocrit and relative blood volume changes during HD, while the final volumes of blood and plasma were calculated from anthropometric data [5].

### Model description

**Fig 1:**
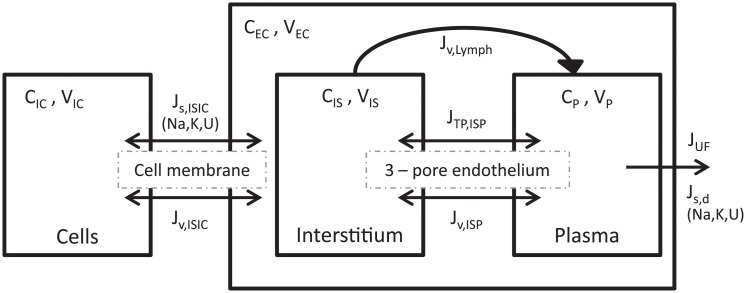
Structure of the model. Each of the three compartments, vascular (*P*), interstitial (*IS*) and intracellular (*IC*), is described in terms of fluid volume (*V*) and concentration of solutes (*C*). Protein (*TP*) mass is divided among interstitial and plasma compartments, and proteins move across the capillary endothelium according to a 3-pore membrane model. Small solute mass is divided between intra- and extracellular spaces; solute concentration in interstitium and plasma is assumed to be constantly offset by 2% at all times. Water is removed by ultrafiltration (*J*_*UF*_) from the plasma compartment, and small solutes are removed by diffusion and convection by the dialyzer. *J*_*v*_—fluid flows; *J*_*s*_—solute flows between compartments *J*_*s*,*d*_—solute flows to dialyzer; *ISIC*—interstitial to intracellular; *ISP*—interstitial to plasma; *U*—urea.

The model describes the distribution of fluid across three compartments, vascular (plasma), interstitial, and intracellular (Fig 1). Proteins (represented by a molecule of the size of albumin) exchange only between interstitium and plasma, while the kinetics of ionic solutes (sodium, potassium) and urea—collectively referred to as *small solutes*–is described with intracellular and extracellular compartments. The underlying assumption is that small solutes traverse the capillary membrane unimpeded, and diffuse so quickly that almost no delay exists between changes of solute’s concentration in plasma and interstitial fluid [14, 16, 17, 25, 26]. The volume of the extracellular space is equal to the sum of plasma and interstitial fluid. The transport of sodium and potassium in the model was based only on mass conservation considerations without taking into account electrodiffusion and the effect of membrane potentials.

The description of the transcapillary transport of fluid and protein implemented in the model was presented in our previous paper, and the related equations are explained and discussed in more detail there [12]. The transport of water and solutes, including proteins, between plasma and interstitium takes place across the pores of the capillary endothelial membrane: large pores (*LP*), small pores (*SP*) and ultrasmall pores (*UP*), according to the 3-pore model [27, 28].

The convention adopted in this article regarding the sign of flows is that flows directed from the intracellular compartment to the interstitium are negative, as are flows from the interstitium to the vascular compartment.

The global fluid flow across the capillary membrane (flow from interstitium to plasma, *ISP*) depends on the net balance of hydrostatic and oncotic pressures between interstitium and plasma, and is equal to the sum of the flows through each type of pore:

$$J_{v,ISP}=Lp\cdot \left\{\left(P_{P}-P_{IS}\right)-\sum_{r=LP,SP,UP}\left[\alpha_{r}\cdot \sigma_{TP,r}\cdot \left(\Pi_{TP,P}-\Pi_{TP,IS}\right)\right]\right\}$$ (1)

where *Lp* is the global filtration coefficient of the capillary membrane, and *α*_*r*_ is the fractional contribution to *Lp* of pore type *r*. *P* is the hydraulic pressure, and *П* the osmotic pressure of proteins (oncotic pressure). The oncotic pressure is calculated from serum total protein concentration using the Landis-Pappenheimer formula [29]. The subscripts are: *P* (plasma), *IS* (interstitium), *TP* (total protein), *r* (pore type) and *s* (solute type). *σ*_*TP*,*r*_ is the Staverman reflection coefficient for proteins in pore *r*, and depends on steric and electrostatic interactions; it is calculated from pore and solute radii [30, 31].

The transport of proteins in the 3-pore system, with diffusive and convective components, takes place according to thermodynamic theory and the Kedem-Katchalsky model, as already seen in other studies [27, 30, 32]. The total transport is given by the sum of individual flows through each pore family with the exclusion of ultrasmall pores, which are impermeable to all solutes because of their size:

$$J_{TP,ISP}=\sum_{r=LP,SP}\left[PS_{TP}\left(C_{TP,P}-C_{TP,IS}\right)+S_{TP,r}\cdot J_{v,r}\cdot C_{TP,m,r}\right]$$ (2)

*PS* is the diffusive permeability surface product for proteins [30], *S* the sieving coefficient (equal to 1 –*σ*), and *J*_*v*,*r*_ the fluid flow across the pore type *r*. *C*_*TP*,*m*,*r*_ is the average concentration of solute inside the membrane for each type of pore and is calculated from the concentrations at the extremities of the pore and the intensity of the flow [30, 33].

The transport of sodium and potassium across the cell membrane (from interstitium to intracellular space, *ISIC*) has two components: passive diffusion and a flow generated by the Na/K pump:

$$\begin{array}{llll} J_{s,ISIC}=w_{s}\left(C_{s,IS}-C_{s,IC}\right)+H_{s}\cdot B\cdot Jp_{MAX} & & & s=Na,K \end{array}$$ (3)

where *w* is the transmembrane diffusivity of each ion, and *Jp*_*MAX*_ is the maximum transfer rate of the pump. The coefficient *B* represents a function that modulates the activity of the pump depending on the concentrations of intracellular sodium and interstitial potassium [34]:

$$B=\frac{C_{K,IS}}{C_{K,IS}+C_{h,K}}\cdot \frac{C_{Na,IC}}{C_{Na,IC}+C_{h,Na}}$$ (4)

Because the pump always transports at the same time two K^+^ ions inside the cell and three Na^+^ ions outside of the cell, the factor *H* is equal to *+2* for potassium and *-3* for sodium and. *C*_*h*,*K*_ and *C*_*h*,*Na*_ are the solutes concentrations for the half-activation of the pump, and their values were taken from literature:10 mmol/L for sodium and 1.5 mmol/L for potassium [34].

The transport of urea is dictated only by passive concentration gradient:

$$J_{U,ISIC}=k_{U}\left(C_{U,IS}-C_{U,IC}\right)$$ (5)

Because proteins are assumed to not cross the cellular membrane, their mass in the intracellular compartment is considered constant.

The transport of water across the cellular membrane is assumed to be function of the changes in the total osmolarity of the compartments, *θ*_*X*_, where the subscript *X* stands for intracellular or interstitial. *θ*_*X*_ can be approximated as:

$$\Theta_{X}=0.93\cdot \left(C_{Na,X}+C_{K,X}+C_{U,X}+C_{TP,X}+\frac{M_{eq,X}}{V_{X}}\right)$$ (6)

where the coefficient 0.93 accounts for reduction in osmotic activity introduced by intermolecular attraction [35]. *M*_*eq*,*X*_ is the *equivalent mass* of all solutes non-described by the model in each compartment, and it is considered constant for the sake of simplicity. The water flow rate across the cell membrane, *J*_*v*,*cell*_, was thus proportional to the osmotic difference at the cellular membrane:

$$J_{v,ISIC}(t)=k_{f}\cdot \left[\Theta_{ic}(t)-\Theta_{is}(t)\right]$$ (7)

The transmembrane water transfer coefficient, *k*_*f*_, incorporates the cell membrane hydraulic filtration coefficient and the factor *R·T*, and its value, *k*_*f*_ = 0.024 L^2^·min^-1^·mmol^-1^, was adapted from the value used in [16, 17] (see Discussion). In Eq 7, it is also assumed that the reflection coefficient of the cell membrane for all solutes is equal to 1 [36].

The flow of removal of solutes by dialysis, *J*_*s*,*d*_, is calculated as:

$$J_{s,d}=J_{v,UF}\cdot C_{s,P}+D_{s}\left(1-\frac{J_{v,UF}}{Q_{b}}\right)\cdot \left(C_{s,P}-C_{s,d}\right)$$ (8)

where *Q*_*b*_ is the blood flow rate in the dialysis circuit, and *D*_*s*_ the dialysance for solute *s* [24]. Dialysances are calculated using concentrations at the inlet and outlet of dialysate line measured at 2 hours into the treatment [37] (Table 1). Concentrations in dialysis fluid for sodium and potassium are shown in Table 1; urea concentration in dialysis fluid was 0 in all sessions.

The complete system of ordinary differential equations for the volume and mass balance is presented in Eq 9:

$$\begin{array}{l} \frac{dV_{P}}{dt}=-J_{v,ISP}+J_{v,Lymph}-J_{v,UF}\\ \frac{dV_{IS}}{dt}=J_{v,ISP}-J_{v,Lymph}-J_{v,ISIC}\\ \frac{dV_{IC}}{dt}=J_{v,ISIC}\\ \frac{dM_{TP,P}}{dt}=-J_{TP,ISP}-J_{TP,Lymph}\\ \frac{dM_{TP,IS}}{dt}=J_{TP,ISP}+J_{TP,Lymph}\\ \frac{dM_{s,EC}}{dt}=-J_{s,ISIC}-J_{s,d}\begin{array}{ccc} & & s=Na,K,U \end{array}\\ \frac{dM_{s,IC}}{dt}=J_{s,ISIC} \end{array}$$ (9)

where *M* represents the mass of each solute, *J*_*v*,*Lymph*_ is the lymphatic flow of fluid, and is equal to *J*_*v*,*Lymph*,0_·[1 + *LS*(*P*_*IS*_(*t*) − *P*_*IS*,0_)], with *LS* being the sensitivity to changes in hydraulic pressure, and *J*_*v*,*Lymph*,*0*_ the flow at steady-state [12, 38]. *J*_*TP*,*Lymph*_ = *C*_*TP*,*IS*_ · *J*_*v*,*Lymph*_ accounts for the bulk flow of proteins toward the vascular compartment with the lymphatic fluid.

The description of water flow across the interface between intracellular and interstitial spaces is an important aspect of the model. Doubts arose concerning the accuracy of this implementation when only few solutes were included as dynamic quantities in Eq 6. In order to test the dependence of *J*_*v*,*ISIC*_ on the number of small solutes described, we implemented a variant of the model with one additional solute (Expanded model). This solute was chosen to have the concentration of chloride, the second most abundant electrolyte in extracellular fluid. Since data on chloride concentration was not available in our samples, general physiological values for intra- and extracellular concentrations were used (110 mmol/L and 4.4 mmol/L for extra- and intracellular compartments, respectively) [39]. The added solute was denoted A, to stress the fact that its inclusion does not represent an attempt to simulate the behavior of an actual ionic species in the patients, but it is an experiment to investigate the characteristics of our model. The transport of solute A was modeled adding two simple diffusion equations to the system of Eq 9:

$$\begin{array}{l} \frac{d\left(C_{A,EC}\cdot V_{EC}\right)}{dt}=-w_{A}\left(C_{A,IS}-\gamma C_{A,IC}\right)-J_{A,d}\\ \frac{d\left(C_{A,IC}\cdot V_{IC}\right)}{dt}=w_{A}\left(C_{A,IS}-\gamma C_{A,IC}\right) \end{array}$$ (10)

where *γ* is the initial ratio between intra- and extracellular concentrations, which assures the equilibrium for solute A at steady state. The diffusivity *w*_*A*_ was assumed to be similar to potassium’s. Dialysance for solute A was assumed to be equal to sodium’s and urea’s; the concentration of solute A in dialysis fluid was chosen so that the diffusive removal gradient would be equal to sodium’s. A new set of the same parameters was estimated for the Expanded model as for the Baseline model, using the same procedure.

### Parameters and initial conditions

The initial value of the state variables (plasma volume, solutes concentrations in plasma) was fitted to the data in a small range centered on the measured values (± 2%), to better accommodate the fit and account for measurement errors in the data. Extracellular and intracellular volumes were taken from the BCM measurements; interstitial volume was calculated as the difference between extracellular and plasma volumes. A list of the parameters fixed a priori is shown in Table 2.

**Table 2:** Fixed parameters of the model.

	Symbol	Value	Reference
Small pores fraction of capillary hydraulic conductivity	*α*_*SP*_	0.6	[40, 41]
Pre-dialytic interstitial to plasma total protein concentration ratio	*R*_*0*_	0.6	See text
Sensitivity of lymph flow	*LS*	0.4	[42]
Urea diffusivity across the cellular membrane (mL/min)	*k*_*U*_	770	[43]
Water transfer coefficient (L^2^·min^-1^·mmol^-1^)	*k*_*f*_	0.024	[16]
Large pores size (Å)		250	[12, 30]
Small pores size (Å)		45	[12, 30]
Ultrasmall pores size (Å)		2	[27, 44]
Protein molecule radius (Å)		35.5	[12, 30]
Urea equilibrium ratio		1	[39, 45]
Potassium equilibrium ratio		35	[39]
Sodium equilibrium ratio		0.0174	[39]

Hydraulic pressures in interstitium and capillary were calculated as explained in [12]: interstitial pressure was calculated with an empirical formula describing its relationship to interstitial volume [46], while capillary pressure was chosen so to assure a null net protein transport across the capillary wall at steady-state. The gradient of hydraulic pressure between interstitial and intracellular fluid was considered null at all times.

The initial concentrations of sodium and potassium in the interstitial fluid were assumed to be lower than in plasma by a constant offset (2%) [39]. Their concentrations in plasma and interstitial fluid were calculated from the total extracellular mass [16]. Initial plasma osmolarity was calculated from the data using an empirical formula [47]:

ΘIS,0=2CNa,P,0+2CK,P,0+CGl,P,0+CU,P,0 (11)

where *C*_*Gl*,*P*,*0*_ is the initial plasma glucose concentration.

The initial intracellular concentrations of sodium and potassium were set at 0.0714 and 35 times the values in plasma, respectively [17, 39]. Urea, being small in size and with neutral electric charge was assumed to have the same initial concentration in all compartments [39]. The pre-dialysis interstitial-to-plasma total protein concentration ratio, *R*_*0*_, was set at 0.6, on the higher boundary of the range of values observed in other studies [11, 48–50]. It was observed that, in this type of model, *R*_*0*_ is strongly related to the intensity of lymphatic refill and its value was thus chosen to obtain lymphatic flows close to those reported in literature [12].

The transmembrane diffusivities of sodium and potassium, which appear in Eq 3, were calculated using Eq 12 and imposing the equilibrium of passive diffusion and active pump flow at steady-state, and depended only on the estimated parameter *Jp*_*MAX*_ and the initial concentrations of sodium and potassium:

$$w_{s}=\frac{Jp_{MAX}\cdot H_{s}\cdot B_{0}}{C_{s,IS,0}-C_{s,IC,0}}$$ (12)

with *H*_*s*_ = -3 and +2 for *s* = Na^+^, and *s* = K^+^, respectively. Their values are shown in Table 3.

**Table 3:** Estimated and calculated parameters of the model (median and quartiles).

	HD1	HD3
**Estimated**		
*α*_*LP*_	0.056 (0.050, 0.059)	0.054 (0.044, 0.062)
*Lp* (mL/min/mmHg)	11.63 (7.9, 14.2)	10.74(6.7, 17.2)
*Jp*_*MAX*_ (mmol/min)	5.52 (3.75, 7.54)	6.08 (3.60, 10.97)
**Calculated**		
*HP*_*c*_ (mmHg)	12.5 (11.3, 14.4)	12.6 (11.3, 14.2)
*w*_*K*_ (mL/min)	24.9 (17.1, 35.5)	25.3 (18.9, 54.9)
*w*_*Na*_ (mL/min)	51.0 (35.0, 71.2)	55.1 (32.5, 101.9)
*PS*_*TP*,*LP*_ (mL/min)	1.58 (1.05, 2.39)	1.38 (0.89, 2.81)
*PS*_*TP*,*SP*_ (mL/min)	0.18 (0.12, 0.22)	0.17 (0.10, 0.27)

*α*_*LP*_—large pores fraction of the hydraulic coefficient; *Lp*—hydraulic coefficient; *Jp*_*MAX*_—Na-K pump maximum exchange rate; *HP*_*c*_—capillary hydraulic pressure; *w*_*X*_ transmembrane diffusive coefficient for ion X; *PS*_*TP*,*Y*_ permeability surface product for total proteins, through pore pathway Y. No significant difference between HD1 and HD3 was found.

### Parameter estimation and statistical methods

**Fig 2:**
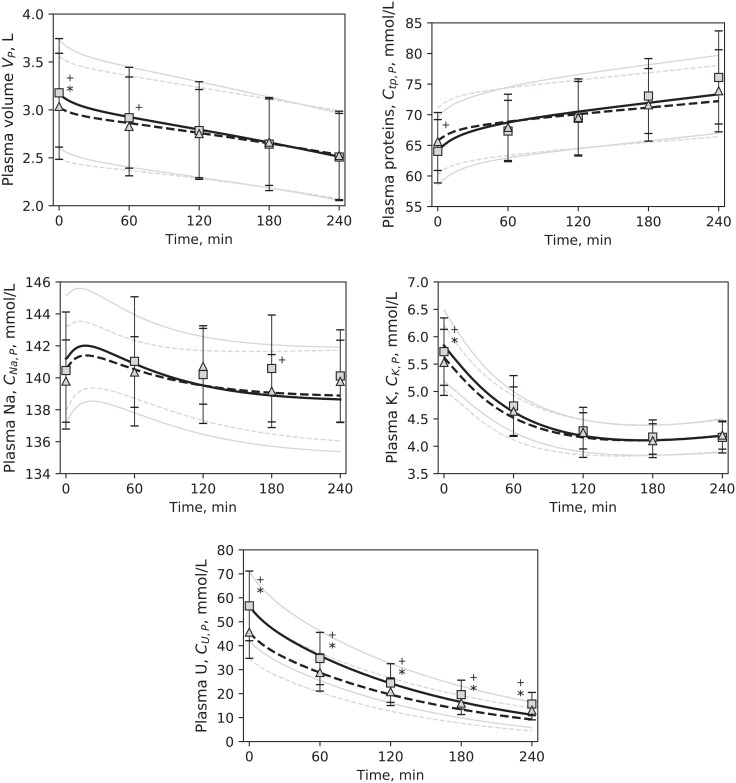
Output of the model. Simulated values (HD1 –continuous line, HD3 –dashed line) compared to clinical data (HD1 –squares, HD3 –triangles), mean values. Error bars and pale lines represent standard deviation for simulation and data, respectively. * p < 0.05 for HD1 vs. HD3 in the simulations. ^+^ p < 0.05 for HD1 vs. HD3 in the data. Differences between simulated profiles were tested at t = 0, 60, 120, 180, 240 minutes. U–urea.

Three unknown parameters of the model were estimated fitting the model to the clinical data (Fig 2): the fraction of large pores in hydraulic conductivity (*α*_*LP*_), the global filtration coefficient (*Lp*) and the whole-body maximum Na/K pump rate (*Jp*_*MAX*_). The range for *α*_*LP*_ and *Lp* was set to 0.001–0.150 and 0.1–20.0 mL/min, respectively; the range for *Jp*_*MAX*_ was large: 0.01–100.00 mmol/min because this parameter is scarcely reported in literature, and the few examples that were found presented widely different values [14, 22, 51].

The estimation of the parameters was carried out with a particle swarm optimization algorithm [52]. The error function RMSE (Root-Mean-Squared Error) included information from five variables (plasma volume, total protein, sodium, potassium and urea concentration):

$$RMSE=\sqrt{\frac{1}{N}\cdot {\left(\sum_{Y}\sum_{i}\frac{Y_{i,SIM}-Y_{i,DATA}}{Y_{i,DATA}}\right)}^{2}}$$ (13)

where *Y* represents each variable used in Eq 13, SIM and DATA refer to model simulation and measured data, respectively, and *i* represents the i-th point in a total of 5 points sampled for each variable. Because only two data points were available for interstitial fluid volume (*ISV*) and intracellular volume (*ICV*), they were not included in the global *RMSE*.

Differences between variables and parameters in different dialysis sessions and versions of the model were assessed using nonparametric statistical tests (Friedman and Wilcoxon tests), because of the relatively small dimension of the sample group and the non-normal distribution of many parameters. Differences between two or more time-dependent variables (such as solute concentration profiles) were usually tested at *t* = 0, 60, 120, 180 and 240 minutes. Numerical values were described as median (quartiles), while average profiles with standard deviations were shown in the figures.

## Results

**Fig 3:**
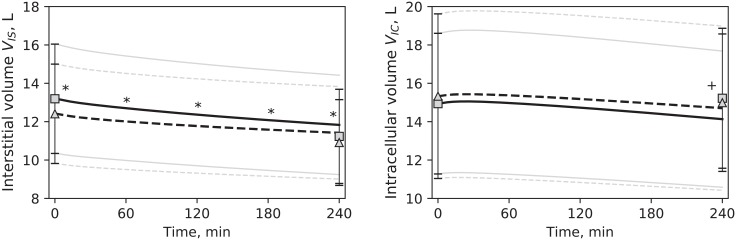
Interstitial volume and intracellular volume. Simulated profiles (HD1 –continuous line, HD3 –dashed line) compared to BCM data (HD1 –squares, HD3 –triangles), mean values. Error bars and pale lines represent standard deviations for simulation and clinical data, respectively * p<0.05 HD1 vs. HD3 in the simulations. ^+^ p < 0.05 for HD1 vs. HD3 in the data. Differences between simulated profiles were tested at t = 0, 60, 120, 180, 240 minutes.

In general, the model offered a good fit to the clinical data, with a global *RMSE* (Eq 13) rarely higher than 5%. Individual *RMSE* values for each of the quantities used in the estimation procedure were likewise small, with the exception of urea, for which the median error was 15–20%; their values are reported in Table 4. The error was similar in all three sessions of the cycle (p > 0.05). Fig 2 shows the fit of the model outputs to the data for HD1 and HD3. Simulated profiles of ISV and ICV are shown in Fig 3, together with the BCM data.

**Table 4:** Deviation of simulated results from clinical data, expressed for each quantity as relative *RMSE* (root mean squared error) and as average error on the profile. Since no statistically significant differences between sessions were found, the median values reported were calculated pooling HD1 and HD3. Note that for interstitial fluid (*ISV*) and intracellular (*ICV*) volumes, *RMSE* was calculated only on one point, and thus simply represents the average error scaled on the data. Global *RMSE* is the output of the objective function in Eq 13. Measurement uncertainties for solutes assays come from the manufacturer of the equipment. For uncertainty on BCM measurements, see [53]).

	RMSE	Average Error
Global	0.039 (0.031, 0.056)	-
Plasma volume	0.017 (0.012, 0.019)	0.049 ± 0.045 L
Total protein**	0.021 (0.016, 0.032)	0.16 ± 0.15 g/dL
Sodium*	0.013 (0.007, 0.016)	1.52 ± 1.28 mmol/L
Potassium*	0.035 (0.026, 0.048)	0.15 ± 0.14 mmol/L
Urea*	0.186 (0.101, 0.230)	3.20 ± 3.0 mmol/L
*ISV*^+^	0.053 (0.018, 0.095)	0.66 ± 0.47 L
*ICV*^+^	0.068 (0.028, 0.082)	1.05 ± 0.98 L

** Measurement uncertainty ~0.2 g/dL.

* Measurement uncertainty ~ 0.2 mmol/L.

^+^ Measurement uncertainty ~ 1–3 L.

The values of the estimated parameters (*α*_*LP*_, *Lp*, *Jp*_*MAX*_) and of other quantities calculated by the model are presented in Table 3. The values of *Lp* showed high scattering, with high interquartile range that covered a large part of the optimization range for the parameter (1–20 mL/min/mmHg). The estimated initial values of the state-variables are not reported: only sodium and potassium were significantly different from the data, but the average difference was negligible (less than 1 and 0.2 mmol/L, respectively).

The flow of fluid through the capillary membrane was partitioned among the different pathways available (ultrasmall, small, and large pores, and lymph) in a similar way to what was already observed in a previous implementation of the model with proteins as the only solute [12]. This was also true for the mass flow of proteins through small and large pores, and lymph.

**Fig 4:**
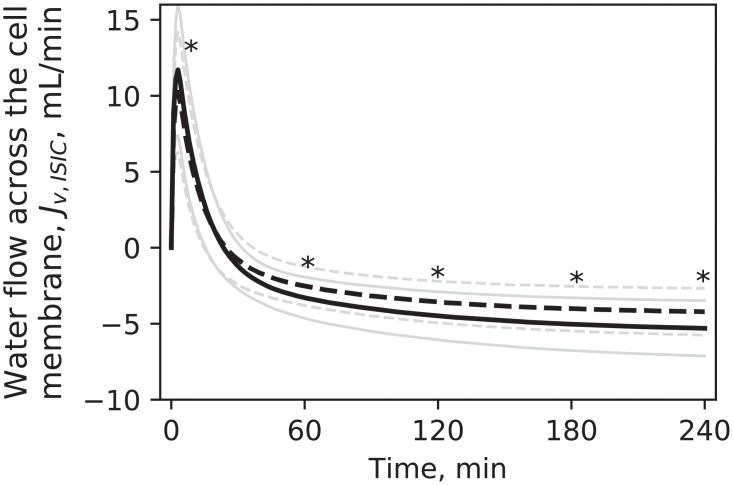
Water flow through the cell membrane. Predicted profiles in HD1 (continuous line) and HD3 (dashed line), mean values. Pale lines are standard deviations. * p < 0.05 for HD1 vs. HD3. Differences were tested at t = 2, 30, 60, 120, 180, 240 minutes.

The simulated flow rate of water through the cell membrane is shown in Fig 4. In all sessions, a spike in flow intensity directed towards the intracellular space was observed when dialysis was initiated (*t* = 0); after about 0.5 h the flow changed direction and water was removed from the cells.

**Fig 5:**
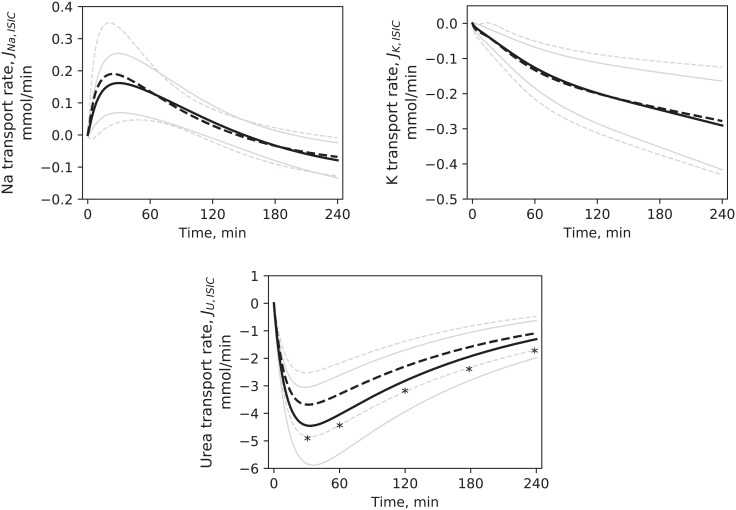
Net small solute transport rates across the cell membrane. Simulated profiles in HD1 (continuous line) and HD3 (dashed line), mean values. Pale lines are standard deviations. * p < 0.05 for HD1 vs. HD3. Differences were tested at t = 30, 60, 120, 180, 240 minutes.

Solute mass flow rates through the cell membrane are shown in Fig 5. For Na and K, flow rate initially equals 0 as the two components of the transport, passive and active, are in equilibrium at steady state. In the case of urea, transcellular transport takes place only through diffusion and the null initial flow rate is caused by urea concentration being at equilibrium between extra- and intracellular space. While for potassium and urea on average only the removal of mass from the intracellular compartment was observed, sodium was transported to the cells for the first 2.5 h, before the flow changed direction for the remaining time of the treatment.

**Fig 6:**
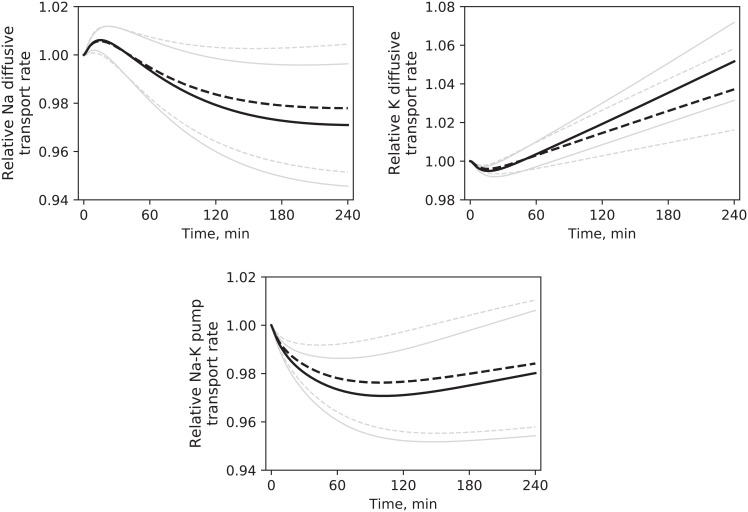
Active and passive transport rate of small solutes across the cell membrane. Relative values for rate of passive diffusion and transport by ATPase pump for sodium and potassium during dialysis in HD1 (continuous line) and HD3 (dashed line), mean values. Pale lines are standard deviations. Profiles are scaled to their respective initial values. HD1 was not different from HD3. Differences were tested at t = 30, 60, 120, 180, 240 minutes.

Fig 6 shows the changes in total cellular transport of Na and K during HD, scaled on the initial value. It should be noted that the direction of passive diffusion is opposite that of pump flow, e.g. sodium diffusion is directed towards the interior of the cells; the opposite is true for potassium.

**Fig 7:**
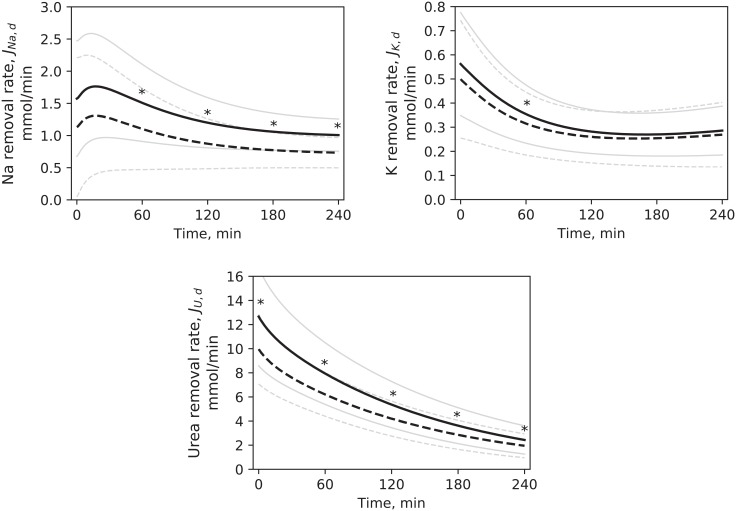
Removal rates of small solutes by dialysis. Simulated profiles in HD1 (continuous line) and HD3 (dashed line), mean values. Pale lines are standard deviations. * p < 0.05 for HD1 vs. HD3. Differences were tested at t = 0, 60, 120, 180, 240 minutes.

Removal rates of sodium, potassium and urea by dialysis were calculated according to Eq 8; the profiles are shown in Fig 7, while the total amounts of solutes removed during the whole treatment are reported in Table 5 and compared with removed mass calculated from dialysate data. Although in some cases the median values of the model underestimated the data, the statistical comparison found no significant difference.

**Table 5:** Total removed mass of small solutes, calculated by the model and from the clinical data. Median and quartiles. No statistical difference was found between data and model results.

		HD1	HD3
Sodium removed (mmol)	Model	297.8 (225.4, 419.2)*	195.9 (162.6, 327.6)
	Data	390.7 (227.2, 548.7)*	235.2 (160.4, 315.6)
Potassium removed (mmol)	Model	81.8 (66.2, 96.2)	64.8 (53.7, 85.0)
	Data	81.0 (45.5, 95.7)	81.0 (50.6, 99.4)
Urea removed (mmol)	Model	1337.9 (1059.8, 1727.9)*	1068.2, (862.9, 1376.0)
	Data	1280.3 (1090.4, 1524.5)*	1047.0 (853.9, 1298.6)

* p < 0.05 for HD1 vs. HD3.

**Fig 8:**
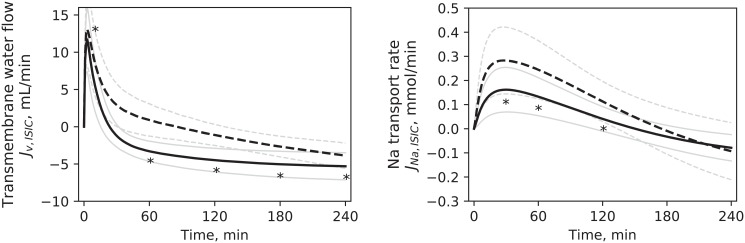
Comparison of simulated flows across the cell membrane. Water (left panel) and sodium (right panel) flow in the Baseline model (continuous line) and Expanded model (dashed line), mean values for HD1. Pale lines are standard deviations. * p < 0.05 for Baseline vs. Expanded.

The results of the estimation of unknown parameters for the Expanded model are shown in Table 6. The simulated profiles of solute concentration were only slightly different from those of the Baseline model, due to the fitting to the same data. The intensity of the various mass and fluid flows changed accordingly to the different values of the estimated parameters, with the biggest differences observed in ionic flows across the cell membrane because of the higher new values of *Jp*_*MAX*_; some examples from HD1 are shown in Fig 8.

**Table 6:** Comparison of estimated parameters between Baseline and Expanded models.

		HD1	HD3
*α*_*LP*_	Expanded	0.046 (0.038, 0.052)*	0.040 (0.035, 0.052)*
	Baseline	0.056 (0.050, 0.059)	0.054 (0.044, 0.062)
*Lp* (mL/min/mmHg)	Expanded	8.14 (6.29, 10.01)*	8.58 (6.27, 12.91)
	Baseline	11.63 (7.9, 14.2)	10.74(6.73, 17.24)
*Jp*_*MAX*_ (mmol/min)	Expanded	11.82 (7.63, 17.34)*^,^^+^	19.74 (8.31, 27.97)*
	Baseline	5.52 (3.75, 7.54)	6.08 (3.60, 10.97)

* p < 0.05 for Baseline vs. Expanded;

^+^ p < 0.05 for HD1 vs. HD3.

## Discussion

The model includes the transport of fluid, proteins and small solutes between three compartments of the human body involved in the disturbed homeostasis during HD. The equations concerning transcapillary transport follow the assumptions of our previous model, presented in [12], which already introduced a detailed view on the components of plasma refilling in HD. The new features are the description of the changes in intracellular volume which occur during an HD session, and the kinetics of small solutes, which directly influences cell membrane water transport. These features appeared more or less recently in others models for the study of HD and HDF (hemodiafiltration), albeit with several differences. The model by Ursino et al described in [15] is based on previous studies by the same authors [16, 17], with improvements and modifications to describe the kinetics of solutes during HDF. Major differences with our model are the lack of the description of protein transport, and of parameters tuning; regarding the latter the authors reported good predictions errors for individual patients just by taking the initial points of model variables from the data [15]. Patient-specific calibration of parameters was instead carried out by Casagrande et al[18], where 3 quantities were estimated by fitting a similar model to clinical measurements of a high number of solutes. The fitting resulted in a marked improvement of the prediction compared to the uncalibrated model, with errors higher for potassium and lower for urea than what we report. Although these models and ours share very similar descriptions of cell membrane water transport, in the formers the active component of the transport of sodium and potassium is not explicated, relying instead on modified diffusion equations to approximate the effect of the Na/K pump (akin to Eq 10). Our implementation was an attempt to increase the physiological basis of the model, without raising much the overall complexity, or incurring in overparametrization. The availability of data on plasma concentration of small solutes was a factor in deciding the extension of the model’s complexity: it was preferred to limit the description of solute kinetics to the species whose data were available for a comparison with the model’s results. As such, only the transport of total protein, sodium, potassium, and urea was described.

The relative errors (expressed as *RMSE*, Table 4) were small for all output variables, with the exception of urea, for which the median error was 15–20%. This high *RMSE* was caused by averaging the small relative errors made on the data points at the beginning of the session, with the high relative errors made on the points at the end. The difference in the magnitude of the relative errors was the result of the decrease of plasma urea concentration from high levels at the start of HD to low values towards the end of the session. As seen in Fig 2, the simulated profile is on the whole close to the clinical data, but the relative error at each individual time point increased with treatment time, reaching up to 50% on the final data point, and affecting the average error for the whole profile.

The prediction error for intracellular and interstitial volumes was somewhat worse than that for plasma volume (Table 4 and Fig 3). Possible reasons include an oversimplified description in the model of the composition of the total osmolarity on the two sides of the cell membrane; this consideration prompted the decision of expanding the model with the addition of more solutes described, as discussed later in this section. However, bioimpedance measurements of fluid compartment are prone to discrepancies between pre- and post-HD values in fluid overloaded patients, as the electrochemical composition of fluid compartments is perturbed by the dialysis treatment [54]. This discrepancy is reflected in our data: the change in total body water during HD, according to BCM data, was 2.0 L (1.7, 2.6) and 1.9 L (1.4, 2.9), for HD1 and HD3, respectively, while it was higher according to the model and to the reported UF volume, 2.8 L (2.2, 3.3) and 2.1 L (1.6, 2.5), for HD1and HD3, respectively (p < 0.05). These differences could also be in part due to the use of whole body bioimpedance spectroscopy that may not accurately capture fluid shifts in arms and legs for which segmental bioimpedance is a better method [55]. Anyway, the distance between intracellular and interstitial simulated volumes and data is comparable to the average accuracy for bioimpedance spectroscopy [53, 56].

**Fig 9:**
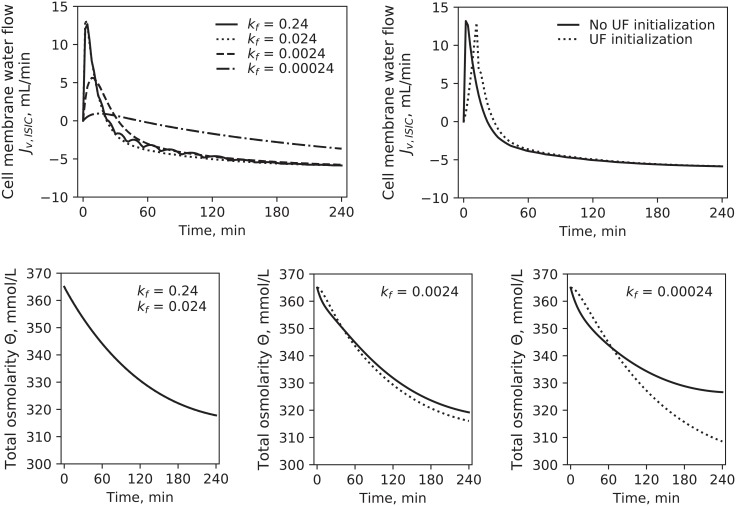
Top left: Transmembrane water flow rate (*J*_*v*,*ISIC*_) calculated with different values of the transmembrane water transfer coefficient *k*_*f*_ (L^2^ min^-1^ mmol^-1^) using Eq 7. Top right: *J*_*v*,*ISIC*_ with UF instantaneously reaching its treatment value (11.3 mL/min) at t = 0 (continuous line) and with UF increasing exponentially from 0: *UF*(*t*) = *λ* (*e*^0.1-t^ − 1). *λ* was chosen so that UF would reach its treatment value in 10 minutes; the coefficient 0.1 was chosen empirically to have a moderately steep increase. Bottom: Interstitial total osmolarity (continuous line) and intracellular total osmolarity (dashed line) calculated with different values of the water transfer coefficient k_f_. The difference between intra- and extracellular osmolarity for *k*_*f*_ = 0.24 and *k*_*f*_ = 0.024 was small and therefore not visible in the chart.

The water flow across the cell membrane, *J*_*v*,*ISIC*_, was proportional to the difference in total osmolarities multiplied by a factor *k*_*f*_, whose value was adapted from literature. The high value of this transmembrane water transfer coefficient was selected to assure that water transport would be fast enough to keep intra- and extracellular osmolarities balanced at all times, in order to avoid the build-up of an excessive osmotic pressure gradient across the cell membrane [16]. This resulted in a spike in water flow towards the cellular compartment when the total extracellular osmolarity changed abruptly due to the instantaneous switching on of dialysis (and thus of small solutes removal). The value of *k*_*f*_ used in our study, 0.024 L^2^ ⋅ min ⋅ mmol^-1^, was 10 times lower than that used in [16] (Fig 9, top left), to avoid the numerical instability in the model solution caused by the higher values; however, the effect of this difference on the profile of *J*_*v*,*ISIC*_ and of total osmolarities was negligible (Fig 9 top left and bottom). Additional simulations were made with an initialization period of 10 minutes, during which ultrafiltration rate and solutes dialysances increased exponentially; this caused the spike in water transport to be delayed but to have a similar peak (Fig 9, top right). These results suggest that the fast inflow of water into the cells was directly associated with the high hydraulic conductivity of the cell membrane and the fast changes of extracellular osmolarity at the beginning of dialysis.

The estimated parameters of fluid and protein transport (*α*_*LP*_ and *Lp*) were, both in the Baseline and Expanded models, not statistically different from the ones found in our previous publication [12], even though the median *Lp* was numerically higher; the lack of significance in the comparison of median values with our previous model might be caused by the relatively high scattering of *Lp* values, especially in the current Baseline model. However, even higher values of *Lp* have been reported in other studies [57, 58].

The unknown parameter introduced in present model was the Na/K ATPase maximum pump rate, *Jp*_*MAX*_, which determines the whole-body transmembrane transport of these small ions across the cell membrane and, indirectly, of water. The equations describing the active transport of sodium and potassium through the cell membrane (Eqs 3 and 4) have not been, until now, implemented in a model for HD; the only example of a similar approach was found in a study describing a single-species potassium model, with a simplified version of Eq 4 [14]. The relative scarcity of examples of whole-body models employing a similar explicit description of the ATPase pump makes it difficult to compare the present results of the estimation of *Jp*_*MAX*_: models available in literature which employ similar equations for sodium and potassium transport describe single cells or have parameters scaled for total surface area of the cells compartment. A two-compartment model of potassium, reporting an estimated value of maximum pump rate of 3.2 mmol/min was presented in [14]; it was however a whole-body average value estimated for the active transport of only one ionic species. An estimation of whole-body *Jp*_*MAX*_ based on physiological measurements of ATPase molecules density in the human skeletal muscle and ox brain ATPase turnover number was reported in [51]. According to this estimation, the total transport capacity in human skeletal muscles amounted to 67 mmol/min, a value much higher than our results of around 6 mmol/min.

The parameter *Jp*_*MAX*_ was the one most affected by the addition to the model of the kinetics of one additional solute (Expanded model). While the transcapillary transport parameters, *α*_*LP*_ and *Lp*, were only slightly different from the one estimated with the Baseline model, *Jp*_*MAX*_ values doubled in the Expanded version, affecting accordingly solute and fluid transport across the cell membrane (Table 6, Fig 8). A possible explanation for the increase of *Jp*_*MAX*_ in the Expanded model is the different way in which the total mass of equivalents in the intra- and extracellular compartments(*M*_*eq*_) was calculated (Eq 6). Because the pre-HD total osmolarity *θ*_*0*_ must be equal to the value estimated via Eq 11, with each additional species modeled *M*_*eq*_ must decrease by the amount of the initial mass of the new solute introduced in the model. For simplicity, it is usually assumed that *M*_*eq*_ in each compartment is constant [16, 21, 22]. While this might be fairly true for proteins and other large solutes, if ionic species are included in this term, the assumption may be far from reality. Not only a fraction of that equivalent mass moves between compartments, but is also dialyzed; as such, extracellular *M*_*eq*_ may decrease during HD, requiring faster transport of solutes from the intracellular compartment to equilibrate the total osmolarities at the two sides of the cell membrane. In the Expanded model, this increased turnover rate is obtained with higher values of *Jp*_*MAX*_. As seen in Fig 9 in the bottom panels, the equilibration between intra- and extracellular osmolarity is highly dependent on the value of the water transfer coefficient *k*_*f*_, which in turn determines the rate of transcellular fluid transport (Fig 9, top-left panel); if the fluid flow is kept constant, the only other way to keep the osmolarity across the cell membrane equilibrated as much as possible is to increase the rate of solute transport. The comparison between Baseline and Extended models highlighted a consequence of approximating the kinetics of total osmolarity that all models based on similar assumptions should take into account.

This theoretical analysis revealed some limitations of our model and suggested that further extensions of the model will need to take into account a higher number of electrolytes for a better estimation of the Na/K ATPase maximum pump rate. Moreover, the equations describing the activation of the Na/K pump were first presented as part of a complex model of cardiac membrane action potential [59], and the impact of neglecting the effect of membrane potential in our simplified version should be further examined. The model also does not account for the presence of osmotically inactive stores of sodium that seem to abound in the skin and muscle [60]. It was recently shown that these tissue stores decrease after HD treatment [61] and the inclusion of this compartment in a future version of the model might be of aid in better understanding the role of these stores. On the other hand, our model demonstrates that the kinetics of sodium ion in plasma during hemodialysis can be well described without the inclusion of non-osmotic stores of sodium. Our approach grants overall a good reproduction of the clinical data, and allows for the estimation of a parameter to individualize the transmembrane transport of sodium and potassium in patients on hemodialysis, during which the equilibrium at the cellular membrane is deeply perturbed. The definition and analysis of parameters such as *Jp*_*MAX*_ is an important step towards a better understanding of the theory of sodium and potassium in non-equilibrium conditions: most of the currently employed models assume a simple equilibrium relationship between ionic concentrations at the sides of cellular membrane to be applied also during nonequlibrium transients. The current attempt to include the activity of Na/K pump, even simplified, in the description of patients with end stage renal disease proposes to apply a relatively simple clinical study for its estimation and comparative analysis of its changes with time on dialysis and its variability in different group of patients. The disturbance of the osmotic equilibrium and small ion concentration across cellular membrane during hemodialysis offers a rather unique possibility to study this active transport at whole body level in clinical studies.

Our model combines the description of the 3-pore structure of the capillary membrane, allowing a more detailed representation of the different components of plasma refilling, with the possibility to simulate the behavior of active and passive flows of sodium and potassium during hemodialysis, all factors intrinsically linked to the distribution of fluid and which are often neglected in models for HD. More research in this direction is needed, because explicitly modelling the action of the Na/K ATPase could provide the clinical tools to quantify with simple parameters its contribution to the preservation of homeostasis in HD patients, and help investigating uremic pathologies linked to impairments in this mechanism [62].
